# Ab Initio Chemical Kinetics for Oxidation of CH_3_OH by N_2_O_4_: Elucidation of the Mechanism for Major Product Formation and Its Relevancy to Tropospheric Chemistry

**DOI:** 10.1021/acs.jpca.4c02433

**Published:** 2024-07-08

**Authors:** Hue-Phuong Trac, Ming-Chang Lin

**Affiliations:** Department of Applied Chemistry, National Yang Ming Chiao Tung University, Hsinchu 30010, Taiwan

## Abstract

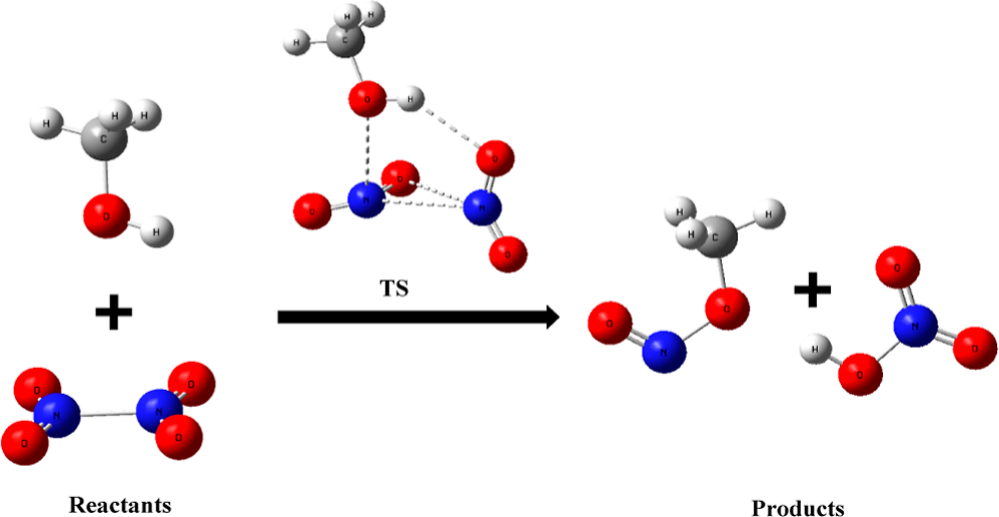

Next to CH_4_, CH_3_OH is the most abundant C_1_ organics in the troposphere. The redox reaction of CH_3_OH with N_2_O_4_ had been shown experimentally to produce CH_3_ONO, instead of CH_3_ONO_2_. The mechanism for the reaction remains unknown to date. We have investigated the reaction by ab initio MO calculations at the UCCSD(T)/6-311+G(3df,2p)//UB3LYP/6-311+G(3df,2p) level. The result indicates that the reaction takes place primarily by the isomerization of N_2_O_4_ to ONONO_2_ through a very loose transition state within the N_2_O_4_–CH_3_OH collision complex with a 14.3 kcal/mol barrier, followed by the rapid attack of ONONO_2_ at CH_3_OH producing CH_3_ONO and HNO_3_. The predicted mechanism for the redox reaction compares closely with the hydrolysis of N_2_O_4_. The computed rate constant, *k*_1_ = 1.43 × 10^–8^ T^1.96^ exp (−9092/T) (200–2000 K) cm^3^molecule^–1^s^–1^, for the formation of CH_3_ONO and HNO_3_ agrees reasonably with available low-temperature kinetic data and is found to be similar to that of the isoelectronic N_2_O_4_ + CH_3_NH_2_ reaction. We have also estimated the kinetics for the termolecular reaction, 2 NO_2_ + CH_3_OH, and compared it with the direct bimolecular process; the latter was found to be 4.4 × 10^5^ times faster under the troposphere condition. On the basis of the known pollution levels of NO_2_, N_2_O_4_, and CH_3_OH, both processes were estimated to be of negligible importance to tropospheric chemistry, however.

## 1 Introduction

CH_3_OH is known to be an important organic pollutant in the troposphere with concentrations averaged to be 600 pptv, next to that of CH_4_.^1^ The origin of CH_3_OH is primarily of terrestrial rather than anthropogenic sources. In the troposphere, CH_3_OH may be removed by reactions with various known radicals such as OH and NO_3_; among them, the destruction by OH is dominant on account of its high reactivity and concentration in the troposphere.^2^ In addition, oxidation by larger nitrogen oxides, N_2_O_*x*_ (*x* = 4 and 5), is also possible under the tropospheric condition as discussed recently by Trac et al.^3^ and by Sarkar and Bandyopadhyay^4^ on the reactivities of N_2_O_4_ and N_2_O_5_ toward NH_3_, respectively. The rate of the former redox process was predicted to be about 100 times faster than that of the latter under the tropospheric condition, although the concentration of N_2_O_4_ is known to be lower than that of N_2_O_5_.^3^

The facile reaction of N_2_O_4_ with CH_3_OH at low temperatures was first reported by Harris and Siegel who noted the disappearance of NO_2_ upon mixing.^5^ Joffe and Gray identified methyl nitrite (CH_3_ONO) and nitric acid as the products of the reaction but not methyl nitrate (CH_3_ONO_2_).^6^ The first kinetic measurement for the N_2_O_4_ + CH_3_OH reaction and several other small alcohols was carried out by Fairlie et al.^7^ at 273–298 K following the NO_2_ decay kinetics by visible light absorption; they reported a negative temperature dependence, obeying the third-order rate law, – d[NO_2_]/dt = 2 *k* [NO_2_]^2^ [CH_3_OH], with *k* = 4.8 × 10^–37^ cm^6^/molecule^2^-s at 298 K. Nikki and co-workers^8^ investigated the reactions of NO_2_ with CH_3_OH and C_2_H_5_OH by FTIR spectroscopy monitoring the growth of RONO which followed the rate law, d[RONO]/dt = *k* [NO_2_]^2^ [ROH]. The rate constant for both alcohol reactions was reported to be the same, *k* = 5.7 × 10^–37^ cm^6^/molecule^2^-s at 298 K. Koda et al.^9^ employed a reactor with spray-mixing to improve the mixing of the two reactants and monitored the kinetics of NO_2_ decay photometrically; they reported the termolecular rate constant to be *k* = 1.79 × 10^–36^ cm^6^/molecule2-s at 294 K. Based on their mechanistic analysis of the NO_2_ decay kinetics, they postulated that the asymmetric N_2_O_4_, or ONONO_2_, might be involved in the reaction giving CH_3_ONO + HNO_3_, instead of the more abundant symmetric N_2_O_4_. More recently, Wojcik-Pastuszka et al.^10^ studied the reactions of N_2_O_4_ with CH_3_OH and several other small alcohols between 293 and 358 K by UV–vis spectroscopy, which allowed them to detect not only NO_2_ and N_2_O_4_ but also the RONO products. The kinetics of these reactions were determined by measurement of NO_2_ decay at 450 nm. They kinetically simulated the NO_2_ decay–time profiles with the mechanism: 2 NO_2_ ⇌ N_2_O_4_ and N_2_O_4_ + ROH ⇌ RONO + HNO_3_. The second-order rate constants for CH_3_ONO formation and its reverse reaction were reported in detail for the CH_3_OH reaction. *To date, the mechanism for the N*_2_*O*_4_*reaction with CH*_3_*OH and other small alcohols remains unknown, however.*

The reactivity of N_2_O_4_ toward NH_3_ and RNH_2_, including methyl amine^3^ (R = CH_3_) and hydrazines^11,12^ [R = NH_2_, CH_3_NH and (CH_3_)_2_N], has been investigated recently by quantum-chemical and statistical-theory calculations in our laboratory. The high reactivity of these reactions was attributed to the unique property of N_2_O_4_, which can undergo isomerization producing a highly reactive isomer ONONO_2_ via a roaming-like transition state (TS) during the course of bimolecular collisions by lengthening of the N–N bond (O_2_N–NO_2_) and rotating one of the O_2_N groups^13^ with barriers below the dissociation limit, ranging from 12.8 to 13.1 kcal/mol depending on collision partners.^3,11−14^ Significantly, the roaming-like TS was also found to exist in the condensed phases including H_2_O^13^ and solid N_2_H_4_,^14^ in which the isomerization reaction produces the reactive ONONO_2_ isomer within the solid lattice producing N_2_H_5_^+^ONO_2_^–^ with a large 70 kcal/mol exothermicity. The half-life of an embedded N_2_O_4_ in solid N_2_H_4_ at 218 K was predicted to be 0.5 s, which could reasonably explain the explosion observed when the N_2_O_4_–N_2_H_4_ solid mixture was warmed up slowly from 77 to 218 K during an experiment.^14^ Parenthetically, it should be mentioned that the conventional isomerization energies for N_2_O_4_ → ONONO_2_ reported in the literature lie in the range of 30–45 kcal/mol,^13^ too high for the hypergolic reactions of N_2_O_4_ with hydrazines to occur.^11,12,14^

In the present study, we investigate the mechanism for the redox reaction of N_2_O_4_ with CH_3_OH producing the experimentally observed products, CH_3_ONO and HNO_3_ by quantum-chemical calculations. If the mechanism of this reaction is similar to those of its isoelectronic analogues, CH_3_NH_2_^3^ and NH_2_NH_2_,^11^ then one would expect the production of CH_3_ONO + HNO_3_ and CH_3_NO_3_ + HONO via a fast and a slow reaction channel, respectively. The significance of CH_3_ONO formation under the tropospheric condition will be examined on the basis of the predicted kinetics.

## 2 Computational Methods

### 2.1 Ab Initio Calculations

The electronic structures of all species involved in the oxidation of CH_3_OH by N_2_O_4_ were optimized with UB3LYP/6-311+G(3df,2p),^15^ including the association step 2NO_2_ + CH_3_OH. To improve energy prediction accuracy, the UCCSD(T)/6-311+G(3df,2p) method was employed with the UB3LYP/6-311 + G(3df,2p) optimized structures. For the heats of reactions forming different products, we have also compared the results obtained with the CCSD(T)/aug-cc-pVTZ//M06-2X/aug-cc-pVTZ method based on the structures optimized with the M06-2X/aug-cc-pVTZ method. Vibrational frequencies of all species involved were calculated at the same level employed for optimization; they were utilized to characterize the stationary points and for ZPE corrections. Unless specified otherwise, all energies cited below are obtained with the UCCSD(T)/6-311+G(3df,2p)//UB3LYP/6-311 + G(3df,2p) method. IRC analyses^16^ have been carried out to confirm the connectivity of TSs with reactants and products. In the present work, all the calculations were performed using the Gaussian 16 program.^17^

### 2.2 Kinetic Calculations

**Figure 1:**
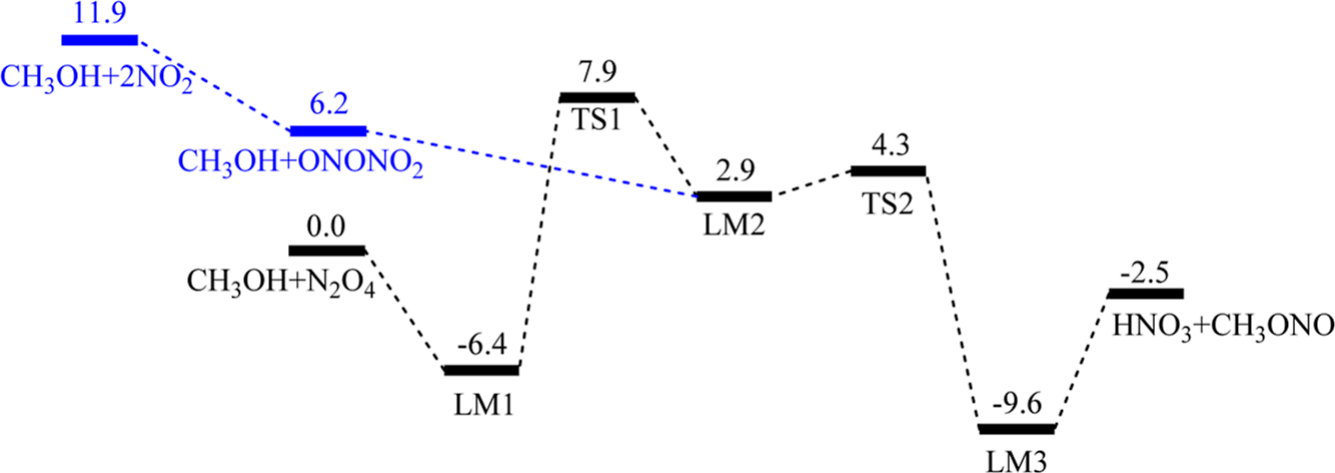
Potential energy profile for the N_2_O_4_ (*D*_2h_) + CH_3_OH reaction calculated at the UCCSD(T)/6-311+G(3df,2p)//UB3LYP/6-311+G(3df,2p) level with ZPE corrections. Relative energies at 0 K are given in kcal/mol.

The kinetics for the reaction of N_2_O_4_ with CH_3_OH was calculated using the Variflex program.^18^ We employed the canonical transition-state theory (TST)^19^ to predict rate constants for a simple exchange reaction, while the RRKM theory^20^ was employed to predict the kinetics of a reaction taking place via a long-lived intermediate by solving the 1-D master equation. We utilized the microcanonical TST^21^ to determine the association rate for a barrierless channel. The potential energy function for the barrierless step N_2_O_4_ + CH_3_OH → N_2_O_4_/CH_3_OH (LM1) was estimated to cover the separation range of N_2_O_4_ and CH_3_OH from 3.19 to13.19 Å with a step size of 0.1 Å at the UB3LYP/6-311 + G(3df,2p) level. The Morse function  represents the minimum energy path obtained by fully optimizing structures along the dissociation coordinate. Here, *D*_e_, *R*, and *R*_e_ have their usual definitions. The calculated Morse function for N_2_O_4_:CH_3_OH (LM1) → N_2_O_4_ + CH_3_OH can be represented with β = 1.38 Å^–1^, and the corresponding value of D_e_ is shown in Figure 1.

## 3 Results and Discussion

### 3.1 PES of the N_2_O_4_ (*D*_2h_) + CH_3_OH Reaction

We have established the PES for the reaction between N_2_O_4_ and CH_3_OH in the gas phase using the UCCSD(T)/6-311+G(3df,2p)//UB3LYP/6-311 + G(3df,2p) level. In Table 1, we first compare the heats of reaction for 2 potential product pairs predicted at the CCSD(T)/6-311 + G(3df,2p) level based on 2 different DFT optimization methods, UB3LYP/6-311 + G(3df,2p) and M06-2X/aug-cc-pVTZ; the result presented in the table indicates that both approaches agree closely, although the former appears to give values of Δ_*r*_H^o^ with a slightly better agreement with available experimental data.

**Table 1:** Comparison of the Predicted Heats of Reaction at 0 K Based on 2 Different Optimization Methods with Experimental Values

reaction	Δ_*r*_H^o^ (kcal/mol)		
	predicted (I)^a^	predicted (II)^a^	literature
N_2_O_4_+CH_3_OH = HNO_3_+CH_3_ONO	–2.5	–2.7	–2.4^28^
N_2_O_4_+CH_3_OH = HONO + CH_3_ONO_2_	–2.5	–2.9	–2.5^28^

a Predicted values (I) by UCCSD(T)/6-311+G(3df,2p)//UB3LYP/6-311+G(3df,2p) and (II) by CCSD(T)/aug-cc-pVTZ//M06-2X/aug-cc-pVTZ.

**Figure 2:**
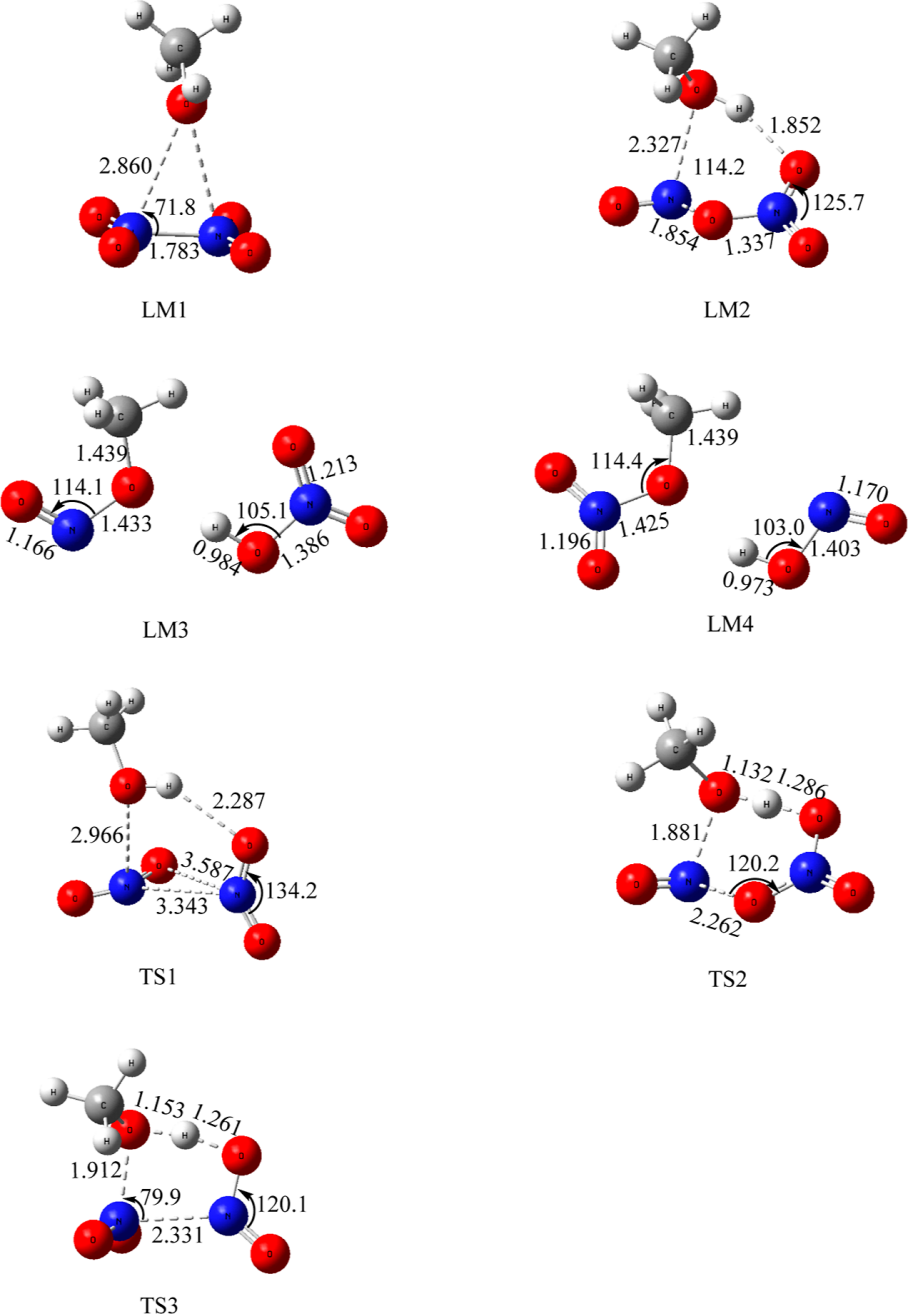
Electronic structures of the key intermediates and TSs involved in the N_2_O_4_ (*D*_2h_) + CH_3_OH reaction optimized at the UB3LYP/6-311+G(3df,2p) level. Bond angles and bond lengths are in degree (°) and angstroms (Å), respectively.

Figure 1 shows the PES for the title reaction predicted using the UCCSD(T)/6-311+G(3df,2p)//UB3LYP/6-311+G(3df,2p) method. Their related structures optimized with the UB3LYP/6-311+G(3df,2p) method are presented in Figure 2. The relative energies and corresponding molecular parameters (vibrational frequencies and moments of inertia) for all involved species are summarized in Tables 2 and S2, respectively.

**Table 2:** Relative Energies of Species in the N_2_O_4_ + CH_3_OH Reaction^a^

	ZPE^b^	UB3LYP/6-311+G(3df,2p)^c^	UCCSD(T)/6-311+G(3df,2p)^c^
N_2_O_4_+CH_3_OH	46.7	–526.031	–525.103
LM1	47.4	–3.1	–6.4
LM2	47.3	6.5	2.9
LM3	47.6	–3.6	–9.6
LM4	47.5	–1.4	–7.3
TS1	44.3	8.7	7.9
TS2	45.4	6.5	4.3
TS3	44.9	25.6	28.1

a The energies are in kcal/mol, relative to that of N_2_O_4_ + CH_3_OH, whose total energy is in Hartree/molecule as given.

b The ZPE in kcal/mol was calculated at the UB3LYP/6-311+G(3df,2p) level.

c The single-point energies are based on electronic structures calculated using UB3LYP/6-311+G(3df,2p) with ZPE corrections.

**Figure 3:**
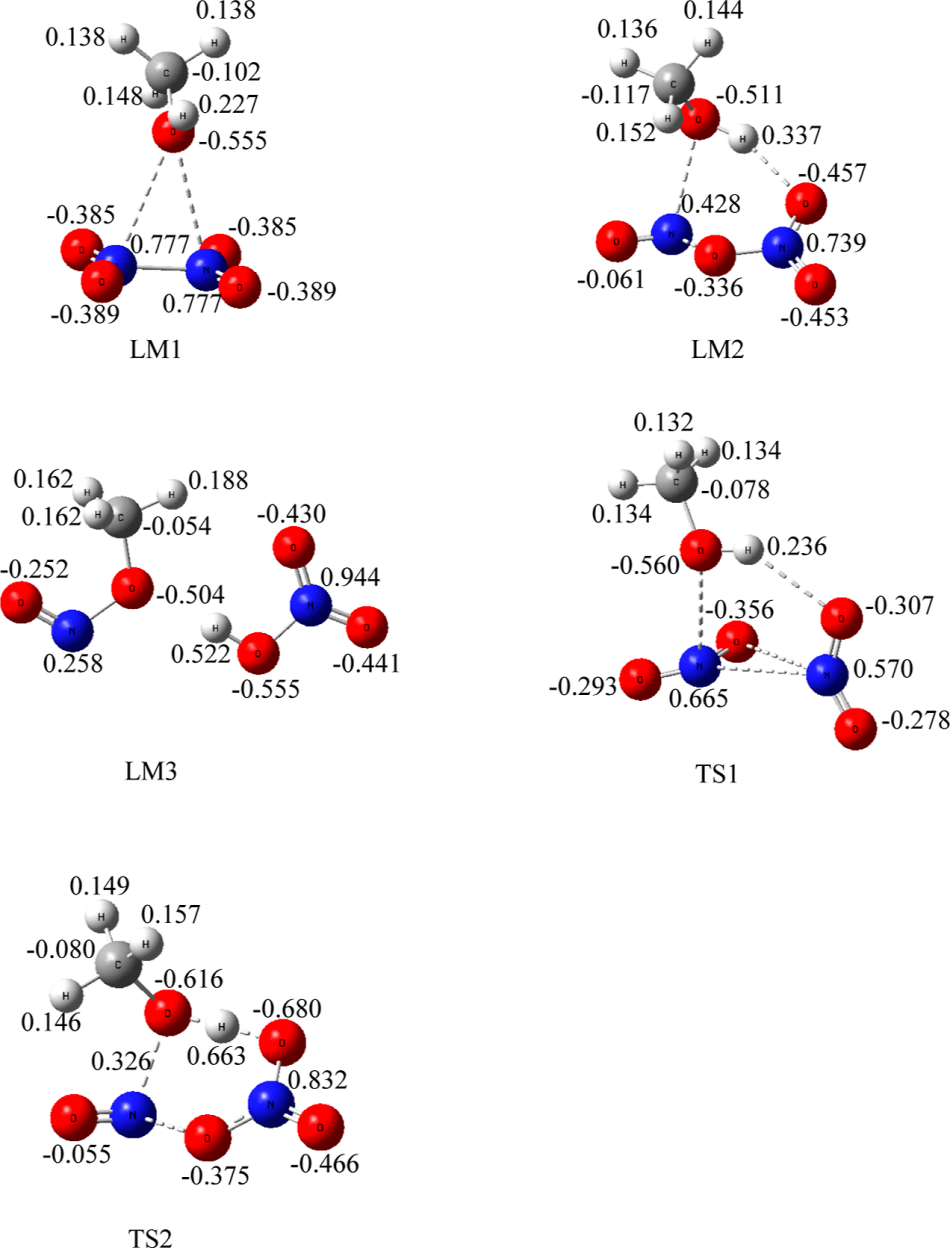
Mulliken charges of species involved in the low-energy reaction path.

In Figure 1, the reaction occurs through the N_2_O_4_:CH_3_OH (LM1) complex with a binding energy of 6.4 kcal/mol, which is slightly more stable than that of the N_2_O_4_/H_2_O complex, 5.0 kcal/mol,^13^ but is very close to its isoelectronic N_2_O_4_/NH_2_NH_2_ complex, 6.7 kcal/mol^11^, and N_2_O_4_/CH_3_NH_2_ complex, 6.8 kcal/mol.^3^ In the present reaction, the LM1 complex can undergo further reaction by lengthening N_2_O_4_’s N–N bond to 3.343 Å concurrently rotating one of its NO_2_ groups at TS1 forming the ONONO_2_/CH_3_OH (LM2) complex, which lies 2.9 kcal/mol above the reactants or 9.3 kcal/mol above LM1. The bond ON–ONO_2_ in the LM2 complex is lengthened to 1.854 Å, which is similar to those in the ONONO_2_/NH_2_NH_2_ complex, 2.282 Å^11^, and ONONO_2_/CH_3_NH_2_ complex, 2.092 Å.^3^ Both are longer than those in *cis-* and *trans*-ON-ONO_2_ isomers, 1.685, and 1.622 Å, respectively,^13^ again suggesting that CH_3_OH can induce ionization of ON-ONO_2_ to form [ON^+^] [NO_3_^–^]. Mulliken charge analysis shows that the NO and NO_3_ group charges are +0.367 and −0.507e, respectively (Figure 3).

In the present case, the same roaming-like TS TS1 exists for the isomerization of N_2_O_4_ to *trans*-ONONO_2_, with a barrier of 7.9 kcal/mol (or 14.3 kcal/mol above LM1), which is again very similar to those of the two isoelectronic systems: N_2_O_4_ + NH_2_NH_2_ 5.9 kcal/mol and N_2_O_4_ + CH_3_NH_2_ 8.0 kcal/mol. From LM2, the NO_3_ group can abstract the H atom of the terminal OH group in CH_3_OH via TS2 with a very small barrier of 1.4 kcal/mol to give the postreaction complex LM3 (HNO_3_:CH_3_ONO) with 9.6 kcal/mol exothermicity. The complex can readily separate to produce the product pair CH_3_ONO + HNO_3_ barrierlessly. To illustrate the effect of CH_3_OH complexation with N_2_O_4_, we have added the potential energy profile for the isomerization of N_2_O_4_ to ONONO_2_ without CH_3_OH predicted by Raghunath and Lin^13^ at the UCCSD(T)/CBS level with Dunning’s correlation consistent basis set (cc-pVXZ, where X = D, T, and Q) based on the optimized structures using UB3LYP/6-311+G(3df). The barrier for the isomerization, 12.8 kcal/mol, is lower than that from N_2_O_4_:CH_3_OH to ONONO_2_:CH_3_OH by 1.5 kcal/mol, which may be attributed to the complexation effect. Other examples on the effect can be found in Table 3.

**Table 3:** Comparison of LM1, TS1, LM2, and TS2 for Various N_2_O_4_ Reactions (Energies in kcal/mol)^a^

reaction	LM1	TS1	LM2	TS2
**N**_**2**_**O**_**4**_**= ONONO**_**2**_		12.8^13^		
**N**_**2**_**O**_**4**_**+ CH**_**3**_**OH = CH**_**3**_**ONO + HNO**_**3**_	–6.4	7.9	–2.9	–4.3
**N**_**2**_**O**_**4**_**+ CH**_**3**_**NH**_**2**_**= CH**_**3**_**NHNO + HNO**_**3**_	–6.8	8.0	2.0	–1.6^3^
**N**_**2**_**O**_**4**_**+ NH**_**3**_**= NH**_**2**_**NO + HNO**_**3**_	–5.3	8.7	4.6	9.9^3^
**N**_**2**_**O**_**4**_**+ H**_**2**_**O = HONO + HNO**_**3**_	–5.0	8.7	2.7	12.6^13^
**N**_**2**_**O**_**4**_**+ N**_**2**_**H**_**4**_**= N**_**2**_**H**_**3**_**NO** + **HNO**_**3**_	–6.7	5.9	–3.8	–4.9^11^
**N**_**2**_**O**_**4**_**+ N**_**2**_**H**_**4**_**= N**_**2**_**H**_**3**_**NO** + **HNO**_**3**_**(in CCl**_**4**_**solvent)**	–5.9	7.4	–8.6	–6.7^29^
**N**_**2**_**O**_**4**_**@HZ**_**23**_**= NO**_**3**_^**–**^**+ N**_**2**_**H**_**3**_**NO + N**_**2**_**H**_**5**_^**+**^	0.0	13.1	–45.0	–43.6^14^
**N**_**2**_**O**_**4**_**+ CH**_**3**_**NHNH**_**2**_**= CH**_**3**_**NHN(H)NO** + **HNO**_**3**_	–6.9	6.3	1.4	1.2^11^
**N**_**2**_**O**_**4**_**+ (CH**_**3**_**)**_**2**_**NNH**_**2**_**= (CH**_**3**_**)**_**2**_**NN(H)NO** + **HNO**_**3**_	–6.0	7.2	0.6	–0.1^12^
**N**_**2**_**O**_**4**_**+ CH**_**3**_**NHNHCH**_**3**_**= CH**_**3**_**N(H)N(CH**_**3**_**)NO** + **HNO**_**3**_	–6.1	9.9	–1.9	–2.1^12^

a UCCSD(T)/6-311+G(3df,2p)//UB3LYP/6-311+G(3df,2p) was employed in refs (3),^11^, and^13^ and this work, CCSD(T)/6-311+G(3df,2p)//B3LYP/6-311+G(3df,2p) was employed for the reaction in refs (12) and^29^ while UCCSD(T)/CBS//UB3LYP/6-311+G(3df) was employed in the N_2_O_4_ isomerization reaction ref (13), and the reaction occurring in N_2_H_4_ solid was predicted with the DFT method in the VASP code.^14^

From LM1, another reaction path involves an H-transfer from CH_3_OH to N_2_O_4_ via a tight five-member-ring TS (TS3) with a 28.1 kcal/mol barrier (or 34.5 kcal/mol above LM1) producing CH_3_ONO_2_ + *trans*-HONO. The reaction was predicted to be exothermic by 2.5 kcal/mol, which is exactly the same as the formation of CH_3_ONO + HNO_3_. This product channel cannot compete with the RTS-like channel producing CH_3_ONO and HNO_3_; it is kinetically unimportant.

In Table 2, the relative energies of all TSs and intermediates computed at UB3LYP/6-311+G(3df,2p) and UCCSD(T)/6-311+G(3df,2p)//UB3LYP/6-311+G(3df,2p) are summarized for a more convenient inspection. In Table 3, we compare the energies of LM1, TS1, LM2, and TS2 relative to each pair of the reactants, from N_2_O_4_ hydrolysis^13^ to N_2_O_4_–hydrazine rocket propellants,^11,12,14^ and to the reactions relevant to tropospheric chemistry.^3^ The implication of these data will be remarked later.

### 3.2 Kinetics of the Reaction

The kinetics of the N_2_O_4_ + CH_3_OH reaction are primarily controlled by TS1 because TS2 lies 3.6 kcal/mol below TS1 with a small 1.4 kcal/mol barrier, producing the postreaction complex with 9.6 kcal/mol exothermicity. Their rate constants were computed with the RRKM theory using the Variflex program, in the 200–2000 K range. For the reaction of N_2_O_4_ with CH_3_OH, the dominant product channel produces CH_3_ONO + HNO_3_, while the second channel is not considered due to its high barrier at TS3. The low-energy reaction path shown in Figure 1 can be given as follows
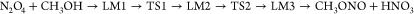


The prereaction complex LM1 with the small 6.4 kcal/mol well is expected to bring about a minor P effect below room temperature as shown in Figure 1S; the formation of CH_3_ONO + HNO_3_ is, however, not affected by pressure as indicated in the figure. Because TS1 (7.9 kcal/mol) is much higher than TS2 (4.3 kcal/mol) and the well depth of LM2 is only 1.4 kcal/mol, as alluded to above, the kinetics of the N_2_O_4_ + CH_3_OH producing CH_3_ONO + HNO_3_ is solely controlled by TS1. The rate expression for the formation of CH_3_ONO + HNO_3_ in cm^3^molecule^–1^s^–1^ is determined by fitting the computed result with the 3 parameter Arrhenius equation at *T* = 200–2000 K
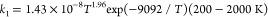


**Figure 4:**
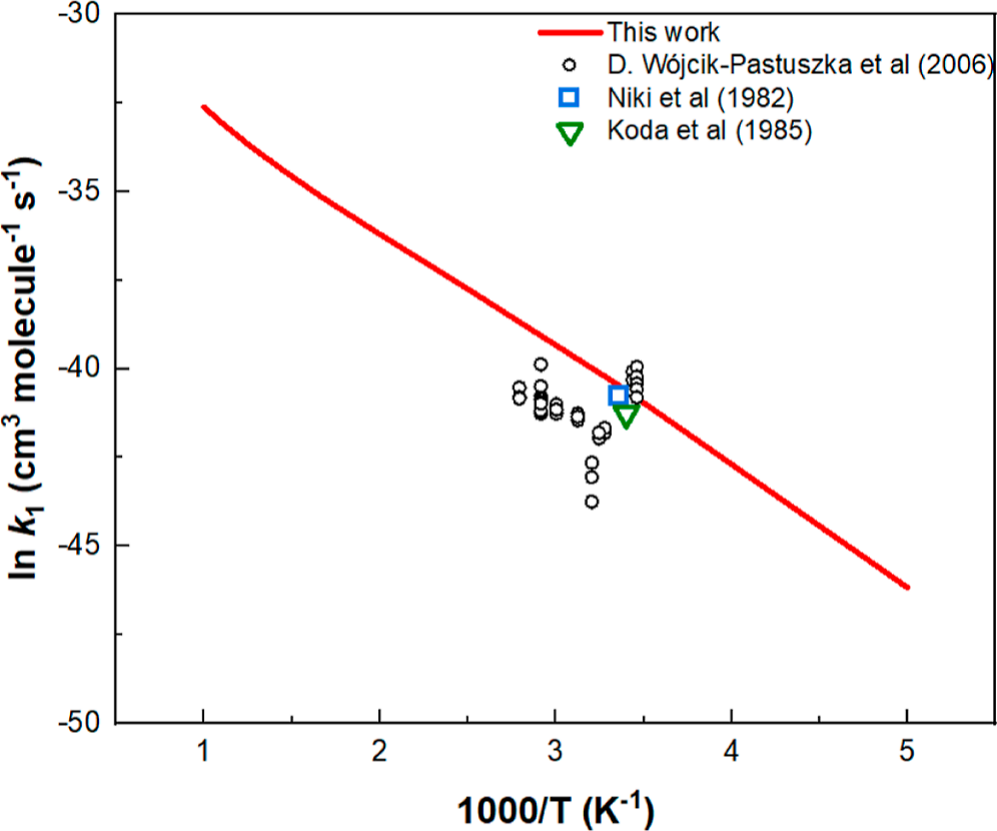
Comparison of the predicted bimolecular rate constant for N_2_O_4_ + CH_3_OH→CH_3_ONO + HNO_3_ reaction with the values evaluated by Wojcik-Pastuszka et al.^10^ through kinetic modeling of NO_2_-time profiles. The third-order rate constants of both Nikki^8^ and Koda^9^ were kinetically modeled by Wojcik-Pastuszka et al.^10^ to give the second-order rate constants as shown.

Figure 4 compares the predicted bimolecular rate constant for CH_3_ONO + HNO_3_ production with the result of Wojcik-Pastuszka et al.^10^ evaluated by kinetic modeling of NO_2_ decay–time profiles using the 4 reaction mechanism including the forward and reverse processes: 2 NO_2_ ⇌ N_2_O_4_ and N_2_O_4_ + CH_3_OH ⇌ CH_3_ONO + HNO_3_ as mentioned in the Introduction. In the figure, we also present the results of Nikki^8^ and Koda,^9^ whose original third-order rate constants were kinetically modeled for N_2_O_4_ + CH_3_OH by Wojcik-Pastuszka et al.^10^ with the 4-reaction mechanism. The agreement between the predicted value and 3 sets of experimental results appears to be reasonable.

**Figure 5:**
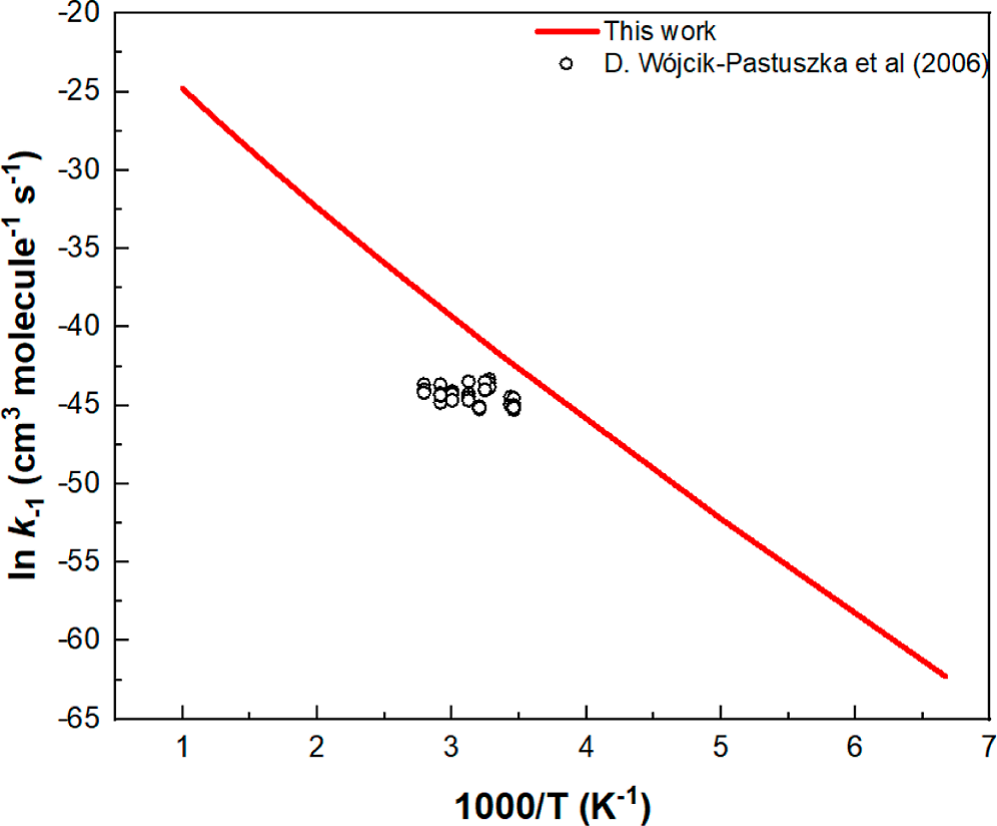
Comparison of the predicted bimolecular rate constant for the reverse reaction CH_3_ONO + HNO_3_→N_2_O_4_ + CH_3_OH with the values evaluated by Wojcik-Pastuszka et al.^10^ through kinetic modeling of NO_2_-time decay profiles.

The modeling of Wojcik-Pastuszka et al.^10^ also gave the rate constant for the reverse reaction, CH_3_ONO + HNO_3_ → N_2_O_4_ + CH_3_OH, *k*_–1_. In Figure 5, we compare the predicted value of *k*_–1_ with those obtained by the modeling based on the 4 reactions. The predicted value exhibits a substantial T dependence reflecting the reverse 10.4 kcal/mol barrier as indicated in Figure 1, whereas the model results showed no T dependence. This may reflect the result of poor initial static mixing of the reactants at low temperatures in a tubular reactor typically employed in a photometric measurement.

Estimation of the Contribution from the Termolecular 2NO_2_ + CH_3_OH Reaction

The relative average concentration of NO_2_ and N_2_O_4_ in the troposphere in ppbv is known to be about 100:7 × 10^–5^.^1^ We, therefore, attempted to estimate the termolecular rate constant for its potential contribution to the oxidation of CH_3_OH under the tropospheric condition. The method employed is similar to the one we used earlier for our quantitative interpretation of the termolecular kinetics for the 2NO_2_ + H_2_O reaction,^22^ which had been reliably measured in laboratories^23^ for comparison. In the present system, we can approximately account for the formation of CH_3_ONO + HNO_3_ by the stepwise mechanism
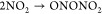
2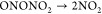-2
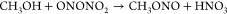
3

The mechanism is similar to that invoked by Koda et al.,^9^ who initially assumed N_2_O_4_ instead of ONONO_2_ as the intermediate. However, based on their analysis of the measured NO_2_ decay kinetics, they concluded that ONONO_2_, rather than N_2_O_4_, was actually involved in the reaction.^9^ Interestingly, this conclusion is consistent with the PES presented in Figure 1 predicted by our high-level quantum calculations.

The steady-state assumption for the unstable ONONO_2_ intermediate leads to the rate for removal of CH_3_OH
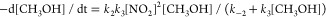
4

The 3 rate constants in eq 4 can be reliably predicted based on the energetics and structures computed at the UCCSD(T)/6-311+G(3df,2p)//UB3LYP/6-311+G(3df,2p) level. Our computed results at atmospheric pressure can be represented by the 3-parameter fitted Arrhenius equations
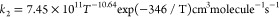

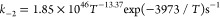

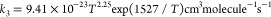


Under the tropospheric condition, it can be shown that *k*_–2_ ≫ *k*_3_ [CH_3_OH]; eq 4 becomes
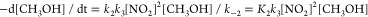
where *K*_2_ = *k*_2_/*k*_–2_ is the equilibrium constant for reaction (2). Take 200 K and 760 Torr, for example, the concentration of CH_3_OH in the troposphere is 9.63 × 10^11^ molecules cm^–3^, *k*_2_ = 2.93 × 10^–14^ cm^3^molecule^–1^s^–1^, *k*_–2_ = 5.41 × 10^6^ s^–1^, and *k*_3_ = 2.56 × 10^–14^ cm^3^ molecule^–1^ s^–1^; therefore, *k*_–2_ = 5.41 × 10^6^ s^–1^ ≫ *k*_3_ x [CH_3_OH] = 2.47 × 10^–2^ s^–1^.

Therefore, at 200 K in the troposphere, we can show that the rate of CH_3_OH reaction by the termolecular mechanism presented above is about 4.4 × 10^5^ times smaller than that of the bimolecular N_2_O_4_ + CH_3_OH reaction as estimated below by taking [NO_2_]/[N_2_O_4_] = 100/7 × 10^–5^,^24^

[bimolecular rate constant; *k*_1_] × [N_2_O_4_] = 8.85 × 10^–21^ × [7 × 10^–5^] = 6.20 × 10^–25^;

[3-step termolecular rate constant; *k*_tf_ ] × [NO_2_]^2^ = 1.40 × 10^–34^ × [100]^2^ = 1.40 × 10^–30^, where the termolecular forward rate constant
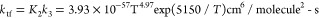


Accordingly, *k*_1_ [N_2_O_4_]/*k*_tf_ [NO_2_]2 = 4.4 × 10^5^ under the tropospheric condition; CH_3_OH can therefore be more effectively removed by the bimolecular N_2_O_4_ reaction, rather than the termolecular process.

### 3.3 Comparison of the Predicted Termolecular Rate Constant with Experimental Data

**Figure 6:**
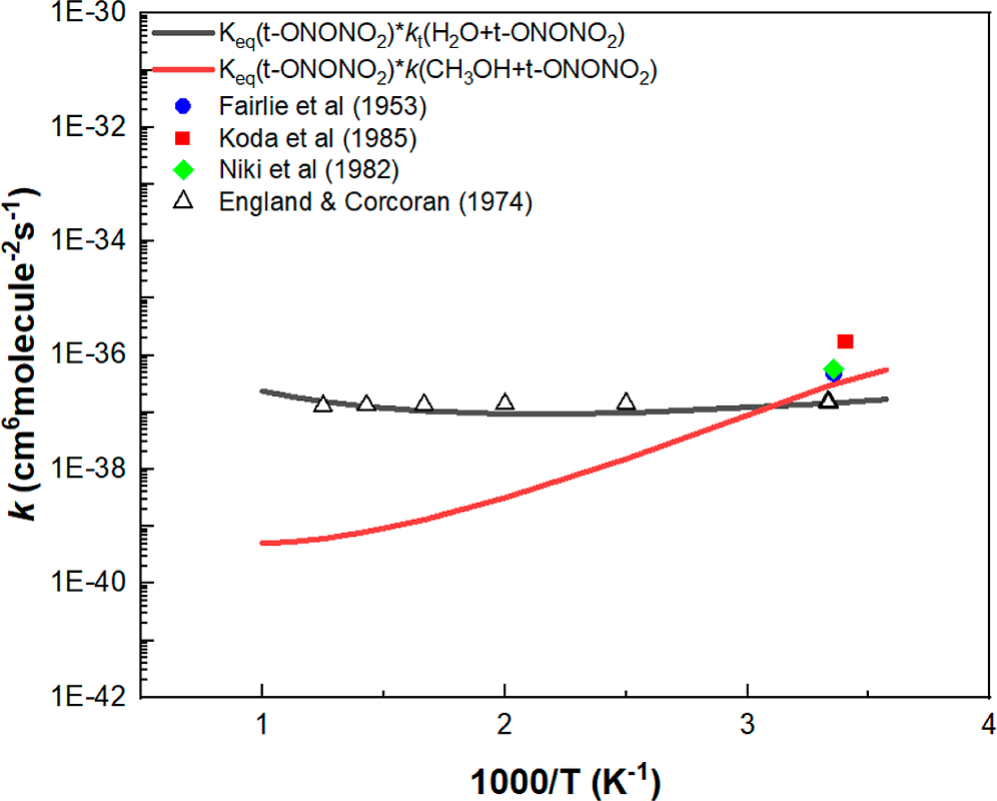
Comparison of the predicted third-order rate constant for 2NO_2_ + CH_3_OH with the experimental values of Fairlie et al.,^7^ Nikki et al.,^8^ and Koda and co-workers,^9^ as well as with that of the analogous 2NO_2_ + H_2_O reaction reported by Zhu et al.^22^ who compared the predicted third-order rate constant with the experimental result of England and Corcoran.^23^

As the termolecular kinetics for 2NO_2_ + CH_3_OH → CH_3_ONO + HNO_3_ had been reported experimentally by Fairlie et al.,^7^ Nikki et al.,^8^ and Koda and co-workers^9^ as aforementioned, we compare the predicted value of *k*_tf_ with the experimental results as shown in Figure 6. The agreement between the theory and the available experimental values is seen to be quite reasonable. In the figure, we also compare the *k*_tf_ for the 2NO_2_ + CH_3_OH reaction with that of the analogous 2NO_2_ + H_2_O → HONO + HNO_3_ reaction measured experimentally by England and Corcoran^23^ together with our previously computed result based on the analogous 3-step mechanism^22^ shown above. In the case of the H_2_O reaction, the agreement between the theory and experiment is seen to be excellent, reflecting the validity of the predicted PES similar to the one shown in Figure 1.

The very different temperature dependences of the termolecular rate constants, as is evident in Figure 6, can be attributed to the strong positive temperature effect on *k*_3_ for the ONONO_2_ + H_2_O reaction (due to its high exit barrier) and the weak T effect on the ONONO_2_ + CH_3_OH reaction as shown in Figure S3 because of its low exit barrier.

### 3.4 Relevancy to the Tropospheric Chemistry

Based on the rate constant *k*_1_ given above for the formation of CH_3_ONO and the concentration levels of NO_2_, N_2_O_4_, and CH_3_OH in the polluted urban atmosphere, [NO_2_] ∼ 2 × 10^11^ molecules cm^–3^,^25^ [N_2_O_4_] ∼ 2 × 10^5^ molecules cm^–3^,^24^ and [CH_3_OH] ∼ 1 × 10^12^ molecules cm^–3^,^26^ we can estimate the effective rate constant for removal of CH_3_OH by N_2_O_4_ at the lower troposphere at 298 K: *k*_1_ × [N_2_O_4_] = 2.7 × 10^–18^ × [1.6 × 10^5^] = 4.4 × 10^–13^ s^–1^. The result suggests that the reaction is too slow to be significant.

## 4 Concluding Remarks

In this study, we have investigated the mechanism for the redox reaction of N_2_O_4_ with CH_3_OH by quantum-chemical calculations. The result of the calculations carried out at the UCCSD(T)/6-311+G(3df,2p)//UB3LYP/6-311 + G(3df,2p) level indicates that the favored reaction path affording the major products CH_3_ONO + HNO_3_ as reported in 1951 by Joffe and Gray^6^ was controlled by the isomerization process forming *trans*-ONONO_2_ from N_2_O_4_ in the presence of CH_3_OH during the bimolecular collision. The highly polar and reactive *trans*-ONONO_2_ rapidly attacks the CH_3_OH molecule producing CH_3_ONO and HNO_3_ via a 6-member-ring TS with a negligible barrier.

For the N_2_O_4_ → ONONO_2_ isomerization via the roaming-like TS within the N_2_O_4_–CH_3_OH complex, the barrier was predicted to be 7.9 kcal/mol above the reactants (or 14.3 kcal/mol from the prereaction complex). This barrier is significantly lower than the typical tight TS for the unimolecular isomerization (∼30–45 kcal/mol).^27^ The predicted rate constant for CH_3_ONO + HNO_3_ formation can be given by *k*_1_ = 1.43 × 10^–8^*T*^1.96^ exp(−9092/*T*) cm^3^molecule^–1^s^–1^ at *T* = 200–2000 K, independent of pressure. The result agrees very closely with that of the isoelectronic reaction N_2_O_4_ + CH_3_NH_2_ as shown in Figure S2.

We have also predicted the kinetics of the 2NO_2_ + CH_3_OH termolecular reaction based on the mechanism employed for the analogous 2NO_2_ + H_2_O → HONO + HNO_3_ reaction.^22^ Comparing our predicted second- and third-order rate constants with available, but scarce, experimental data in the literature for CH_3_OH reactions with N_2_O_4_ and NO_2_, respectively, the agreement between theory and experiments by and large appears to be reasonable.

The unusual reaction mechanism revealed from this series of studies, starting from the hydrolysis of N_2_O_4_ in the gas phase and in the H_2_O solution^13^ to the hypergolic ignition of N_2_O_4_ in contact with hydrazine propellants,^11,12,14^ and the processes relevant to the tropospheric chemistry involving potential pollutants such as NH_3_^3^ and CH_3_OH indicate that the redox reactions occur via prereaction complexes with about 5 ± 1 kcal/mol binding energies which have only a negligible kinetic consequence except at low temperatures. The results were summarized in Table 3. The redox process starts from the isomerization of the symmetric N_2_O_4_ to *trans*-ONONO_2_ via a very loose, roaming-like TS1, lying above the complex well at about 14 ± 2 kcal/mol. The highly polar ONONO_2_ isomer is much more reactive than N_2_O_4_ toward the collision partner as is evident from the small TS2 barrier above LM2 in the present case (see Figure 1). However, the barrier at TS2 above LM2 for H_2_O was predicted to be about 10 kcal/mol, which may be compared with that of its isoelectronic reaction with NH_3_, 5.3 kcal/mol, and the very small value of 1.4 kcal/mol for CH_3_OH, reflecting entirely the strength of the bond to be broken by the abstraction reaction of the NO_3_ group producing HNO_3_.

In view of the fact that both N_2_O_4_ and CH_3_OH are known pollutants in the lower troposphere, we have examined the potential effect of the formation of CH_3_ONO and HNO_3_ from their reaction based on the predicted kinetics given above. Based on the known concentration levels of N_2_O_4_^24^ and CH_3_OH,^26^ the rate of their reaction at 298 K was found to be too slow to be relevant to the troposphere chemistry.

## Supporting Information Available

Studying the impact of pressure on the N_2_O_4_ + CH_3_OH reaction and comparing rate constants for product formation in the N_2_O_4_ reaction with CH_3_OH and CH_3_NH_2_, as well as comparing bimolecular rate constants predicted for ONONO_2_ reactions with CH_3_OH and H_2_O (PDF)

## References

[ref1] Heikes B. G.; Chang W.; Pilson M. E. Q.; Swift E.; Singh H. B.; Guenther A.; Jacob D. J.; Field B. D.; Fall R.; Riemer D.; et al. Atmospheric Methanol Budget and Ocean Implication. Global Biogeochem. Cycles 2002, 16, 1–23. 10.1029/2002gb001895.

[ref2] Speight J. G. Environmental Organic Chemistry for Engineers; Butterworth-Heinemann: Oxford, U.K., 2017.

[ref3] Trac H. P.; Le Huyen T.; Lin M.-C. A Computational Study on the Redox Reactions of Ammonia and Methylamine with Nitrogen Tetroxide. J. Phys. Chem. A 2020, 124, 9923–9932. 10.1021/acs.jpca.0c08665. 33201710

[ref4] Sarkar S.; Bandyopadhyay B. Reaction between N_2_O_5_ and NH_3_ under Tropospheric Conditions: A Quantum Chemical and Chemical Kinetic Investigation. J. Phys. Chem. A 2020, 124, 3564–3572. 10.1021/acs.jpca.0c00580. 32295342

[ref5] Harris L.; Siegel B. M. Reactions of Nitrogen Dioxide with Other Gases. J. Am. Chem. Soc. 1941, 63, 2520–2523. 10.1021/ja01854a061.

[ref6] Yoffe A. D.; Gray P. 325. Esterification by dinitrogen tetroxide. J. Chem. Soc. 1951, 1412–1414. 10.1039/jr9510001412.

[ref7] Fairlie J.; A M.; Carberry J. J.; Treacy J. C. A Study of the Kinetics of the Reaction between Nitrogen Dioxide and Alcohols. J. Am. Chem. Soc. 1953, 75, 3786–3789. 10.1021/ja01111a054.

[ref8] Niki H.; Maker P. D.; Savage C. M.; Breitenbach L. P. An FTIR Study of the Reaction between Nitrogen Dioxide and Alcohols. Int. J. Chem. Kinet. 1982, 14, 1199–1209. 10.1002/kin.550141104.

[ref9] Koda S.; Yoshikawa K.; Okada J.; Akita K. Reaction Kinetics of Nitrogen Dioxide with Methanol in the Gas Phase. Environ. Sci. Technol. 1985, 19, 262–264. 10.1021/es00133a008. 22296015

[ref10] Wójcik-Pastuszka D.; Gola A.; Ratajczak E. Gas Phase Kinetics of the Reaction System of 2NO_2_ ↔ N_2_O_4_ and Simple Alcohols between 293–358 K. Polym. J. Chem. 2005, 79, 1301–1313.

[ref11] Trinh L. H.; Raghunath P.; Lin M. C. Ab Initio Chemical Kinetics for Hypergolic Reactions of Nitrogen Tetroxide with Hydrazine and Methyl Hydrazine. Comput. Theor. Chem. 2019, 1163, 112505 10.1016/j.comptc.2019.112505.

[ref12] Trinh L. H.; Raghunath P.; Lin M. C. Ab Initio Chemical Kinetics for Nitrogen Tetroxide Reactions with 1,1- and 1,2-Dimethylhydrazines. Propellants, Explos., Pyrotech. 2020, 45, 1478–1486. 10.1002/prep.201900426.

[ref13] Putikam R.; Lin M. C. A Novel Mechanism for the Isomerization of N_2_O_4_ and Its Implication for the Reaction with H_2_O and Acid Rain Formation. Int. J. Quantum Chem. 2018, 118, 1–10. 10.1002/qua.25560.

[ref14] Raghunath P.; Lin M. C. Isomerization of N_2_O_4_ in Solid N_2_H_4_ and Its Implication for the Explosion of N_2_O_4_–N_2_H_4_ Solid Mixtures. J. Phys. Chem. C 2018, 122, 23501–23505. 10.1021/acs.jpcc.8b07030.

[ref15] Becke A. D. Density-functional Thermochemistry. III. The Role of Exact Exchange. J. Chem. Phys. 1993, 98, 5648–5652. 10.1063/1.464913.

[ref16] Gonzalez C.; Schlegel H. B. Improved Algorithms For Reaction Path Following: Higher-order Implicit Algorithms. J. Chem. Phys. 1991, 95, 5853–5860. 10.1063/1.461606.

[ref17] Frisch M. J.; Trucks G. W.; Schlegel H. B.; Scuseria G. E.; Robb M. A.; Cheeseman J. R.; Scalmani G.; Barone V.; Petersson G. A.; Nakatsuji H.; Gaussian 16. Revision C.01; Gaussian Inc.: Wallingford, CT, 2016.

[ref18] Klippenstein S.; Wagner A.; Dunbar R.; Wardlaw D.; Robertson S. Variflex, version 1.00; Argonne National Laboratory: Argonne, IL, 1999.

[ref19] Steinfeld J. I.; Francisco J. S.; Hase W. L. Chemical Kinetics and Dynamics; Prentice Hall: Upper Saddle River, N.J., 1999.

[ref20] Wardlaw D. M.; Marcus R. A. RRKM Reaction Rate Theory for Transition States of Any Looseness. Chem. Phys. Lett. 1984, 110, 230–234. 10.1016/0009-2614(84)85219-7.

[ref21] Wardlaw D. M.; Marcus R. A.. In On the Statistical Theory of Unimolecular Processes; Prigogine I., Rice S. A., Eds.; Plenum Press: New York, 1988; Vol. 70, pp 231–263.

[ref22] Zhu R. S.; Lai K.-Y.; Lin M. C. Ab Initio Chemical Kinetics for the Hydrolysis of N_2_O_4_ Isomers in the Gas Phase. J. Phys. Chem. A 2012, 116, 4466–4472. 10.1021/jp302247k. 22506560

[ref23] England C.; Corcoran W. H. Kinetics and Mechanisms of the Gas-Phase Reaction of Water Vapor and Nitrogen Dioxide. Ind. Eng. Chem. Fundam. 1974, 13, 373–384. 10.1021/i160052a014.

[ref24] Kamboures M. A.; Raff J. D.; Miller Y.; Phillips L. F.; Finlayson-Pitts B. J.; Gerber R. B. Complexes of HNO_3_ and NO_3_– with NO_2_ and N_2_O_4_, and Their Potential Role in Atmospheric HONO Formation. Phys. Chem. Chem. Phys. 2008, 10, 6019–6032. 10.1039/b805330h. 18825290

[ref25] Cooper M. J.; Martin R. V.; Hammer M. S.; Levelt P. F.; Veefkind P.; Lamsal L. N.; Krotkov N. A.; Brook J. R.; McLinden C. A. Global Fine-scale Changes in Ambient NO_2_ during COVID-19 Lockdowns. Nature 2022, 601, 380–387. 10.1038/s41586-021-04229-0. 35046607 PMC8770130

[ref26] Bates K. H.; Jacob D. J.; Wang S.; Hornbrook R. S.; Apel E. C.; Kim M. J.; Millet D. B.; Wells K. C.; Chen X.; Brewer J. F.; et al. The Global Budget of Atmospheric Methanol: New Constraints on Secondary, Oceanic, and Terrestrial Sources. J. Geophys. Res. 2021, 126, 1–23. 10.1029/2020jd033439.

[ref27] Pimentel A. S.; Lima F. C. A.; da Silva A. B. F. The Isomerization of Dinitrogen Tetroxide: O_2_N– NO_2_ → ONO– NO_2_. J. Phys. Chem. A 2007, 111, 2913–2920. 10.1021/jp067805z. 17388577

[ref28] Burcat A.; Ruscic B. Third Millennium Ideal Gas and Condensed Phase Thermochemical Database for Combustion with update from Active Thermochemical Tables; Argonne National Laboratory: Argonne, Illinois, U.S.A. and Technion-IIT, Haifa, Israel, 2005.

[ref29] Trinh L. H.; Raghunath P.; Lin M. C. Quantum Chemical Modeling of Spontaneous Reactions of N_2_O_4_ with Hydrazines in CCl_4_ Solution at Low Temperature. Comput. Theor. Chem. 2020, 1188, 112951 10.1016/j.comptc.2020.112951.

