# Supplementary material for: Ab Initio Chemical Kinetics for Oxidation of CH3OH by N2O4: Elucidation of the Mechanism for Major Product Formation and Its Relevancy to Tropospheric Chemistry
Source: J Phys Chem A. 2024 Jul 8;128(28):5548–55. doi: 10.1021/acs.jpca.4c02433 (PMC11264261; doi:10.1021/acs.jpca.4c02433)
Supplement: Supplementary file 1 — jp4c02433_si_001.pdf [file jp4c02433_si_001.pdf]

Supporting Information

Ab Initio Chemical Kinetics for Oxidation of CH_3OH by N_2O_4: Elucidation of the Mechanism for Major Product Formation and its Relevancy to Tropospheric Chemistry

Hue-Phuong Trac and Ming-Chang Lin*
Department of Applied Chemistry, National Yang Ming Chiao Tung University, Hsinchu 300, Taiwan.

1. Pressure effect on the N_2O_4 + CH_3OH reaction

The effect of pressure on the reaction based on the predicted mechanism: N_2O_4 + CH_3OH → LM1 → TS1 → CH_3ONO + HNO_3 has been computed with the Variflex code. The calculated rate constant for the disappearance of CH_3OH is weakly P-dependent due to the 6.4 kcal/mol LM1 well as shown in Fig. S1, while the production of CH_3ONO via the 7.9 kcal/mol barrier at TS1 is P-independent as indicated by the results predicted at 7.6, 760 and 7600 Torr N_2 pressure. The product formation rate constant has been compared with available experimental values in Fig. 4.

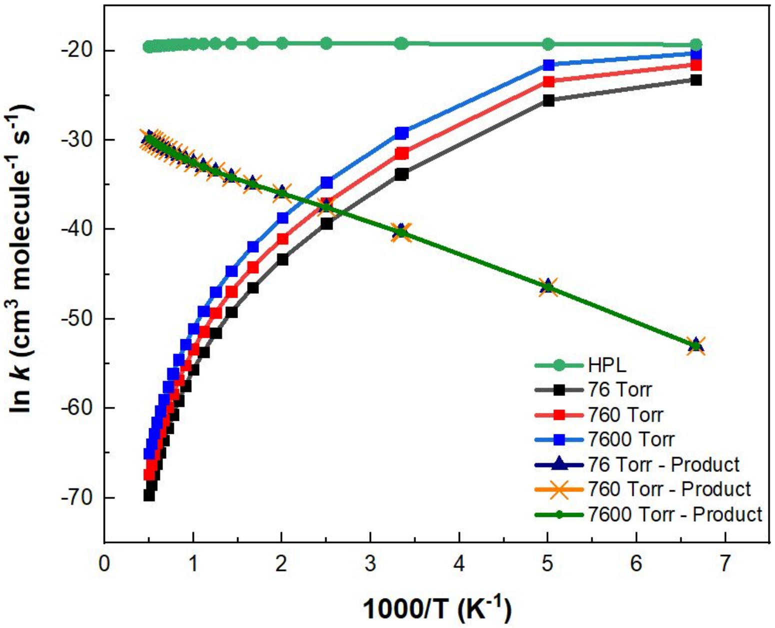

Figure S1. Comparison of calculated rate constants at different pressures on the N_2O_4 (D_{2h}) + CH_3OH reaction in the range of temperature 150-2000 K. The CH_3ONO + HNO_3 product formation is P-independent.

2. **Comparison of the rate constants for product formation in the N_2O_4 reaction with CH_3OH and CH_3NH_2**

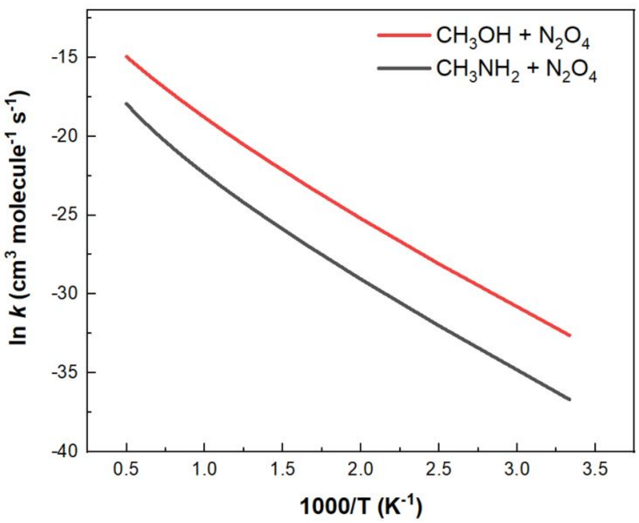

Figure S2. Comparison of the bimolecular rate constants predicted for N_2O_4 reactions with the two isoelectronic molecules CH_3OH and CH_3NH_2.

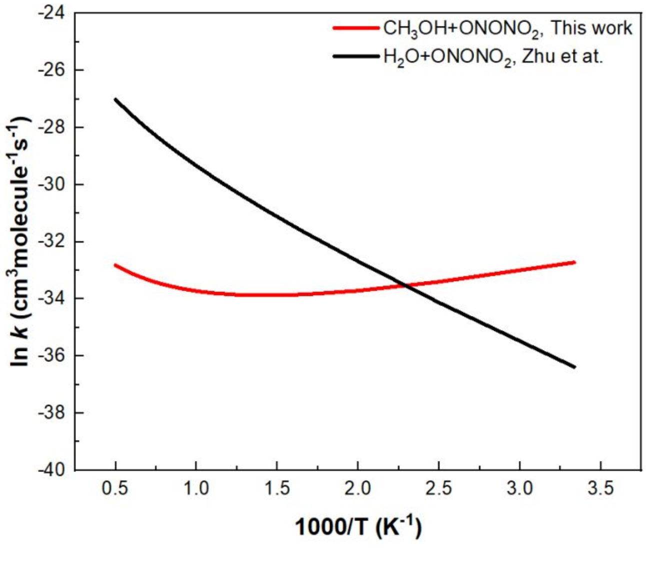

Figure S3. Comparison of the bimolecular rate constants predicted for $ONONO_2$ reactions with CH_3OH and H_2O.
